# Morphometric and topographic evaluations of principal nutrient foramina of the ilium

**DOI:** 10.55730/1300-0144.5579

**Published:** 2022-11-03

**Authors:** Bahattin PASLI, Helin YÜCEDAĞ GÜNDOĞDU, Tuğçe TAŞKINDERE ABBASOĞLU, Burcu ERÇAKMAK GÜNEŞ

**Affiliations:** Department of Anatomy, Faculty of Medicine, Hacettepe University, Ankara, Turkey

**Keywords:** Nutrient foramina, ilium, hip bone, pelvic brim

## Abstract

**Background/aim:**

Nutrient arteries pass through the nutrient foramina and reach the spongious part or medullary cavity of the bones. The arterial blood supply and nutrient foramina of the ilium have not been well described in the literature. The aim of this study is to define the numbers and localizations of nutrient foramina of the ilium on the internal surface in accordance with the pelvic brim, as well as the external surface, and furthermore the distances between these foramina with certain anatomical landmarks.

**Materials and methods:**

This study was performed on 57 dry hip bones. The foramina on the external and internal surfaces of the ilium were evaluated separately. The external surface of the ilium was divided into 4 regions in accordance with the gluteal lines to determine the localizations of the nutrient foramina. The foramina on the internal surface were evaluated according to whether they were above or below the pelvic brim. The diameters of the foramina and their nearest distances to certain anatomical landmarks were measured. Furthermore, the risk area of the region above the pelvic brim was determined.

**Results:**

The foramina that were found on the external surface of the ilium were distributed mainly between anterior and inferior gluteal lines (42 foramina-37.17%) and below the inferior gluteal line (57 foramina-50.44%). On the internal surface, 54 nutrient foramina were found above the pelvic brim and 81 were found below.

**Conclusion:**

The localizations and configurations of all nutrient foramina on the ilium were evaluated morphometrically for the first time.

## 1. Introduction

Nutrient arteries are the major blood supplies of the bones [1–3]. They pass through the nutrient foramina and reach the spongious part or medullary cavity of the bones [1,4]. These arteries are significant during the early embryonic stages of ossification [5–9]. During the transformation of cartilage tissue to bony tissue, nutrient vessels pass through the periosteum layer and form nutrient foramina which provides a vascular conduit between the exterior and interior parts of the bones [7].

Ilium has more nutrient foramina than long bones and the diameters vary widely [9–12]. The ones with larger diameters are defined as principal foramina [10]. Although there are many studies found in the literature about nutrient foramina, most of them are related to long bones [9,11,12]. The studies found in the literature that are related to the foramina of the hip bone are few in number. These studies are describing the nutrient foramina located on the internal surface of the ilium above the pelvic brim [10,13]. No study has been reported about the foramina located below the pelvic brim and the external surface of the ilium.

The aim of this study is to describe the numbers and localization of nutrient foramina on the internal surface in accordance with the pelvic brim and the external surface of the ilium, and to measure the distances between these foramina with certain anatomical landmarks.

## 2. Materials and methods

This study was performed on 57 dry hip bones of unknown age and gender that belong to the collection of dry bones in Hacettepe University, Faculty of Medicine, Department of Anatomy. Six of the hip bones were excluded from the external surface evaluation due to the deformity of the certain landmarks, but all bones were included in the internal surface evaluation as deformed landmarks did not cause any problems at internal measurements. The more prominent nutrient foramina were evaluated due to the presence of the large number of accessory foramina in the hip bone.

### 2.1. External surface

**Figure 1:**
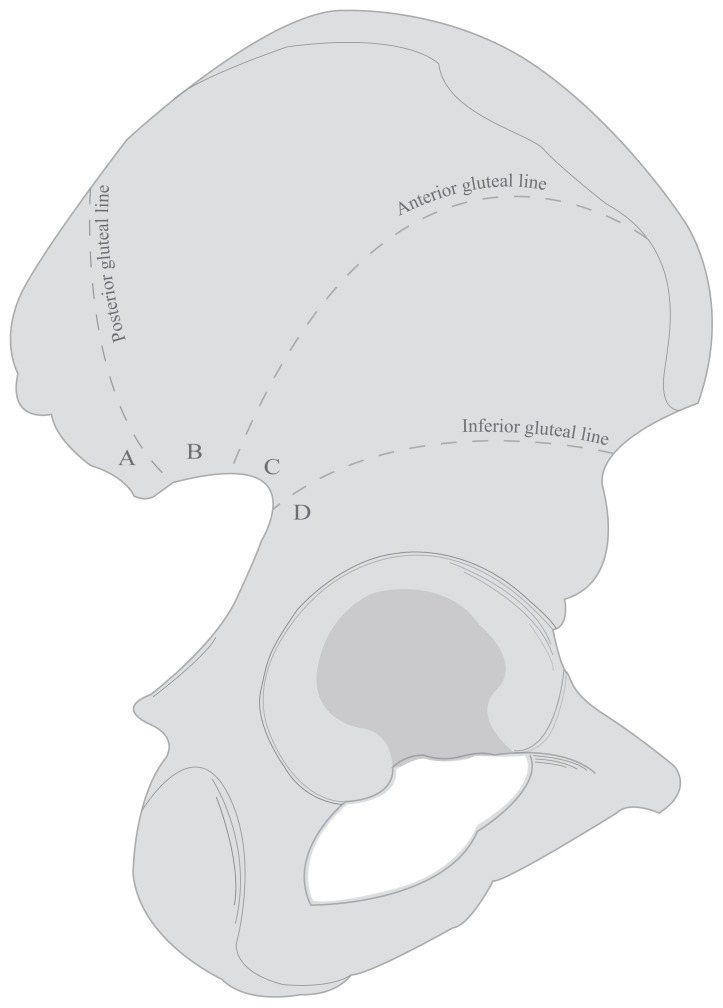
Illustration of division into 4 regions according to the posterior-anterior-inferior gluteal lines (Region A: Above the posterior gluteal line; Region B: Between the posterior and anterior gluteal lines; Region C: Between the anterior and inferior gluteal lines; Region D: Below the inferior gluteal line).

The external surface of the ilium was divided into 4 regions according to the posterior-anterior-inferior gluteal lines and the localizations of the nutrient foramina according to these regions were determined. The gluteal lines are useful anatomical landmarks for demarcating the external surface and to determine the localization of the foramina. Regions were determined as; the area above the posterior gluteal line as Region A, the area between posterior and anterior gluteal lines as Region B, the area between anterior and inferior gluteal lines as Region C and the area below the inferior gluteal line as Region D (Figure 1).

**Figure 2:**
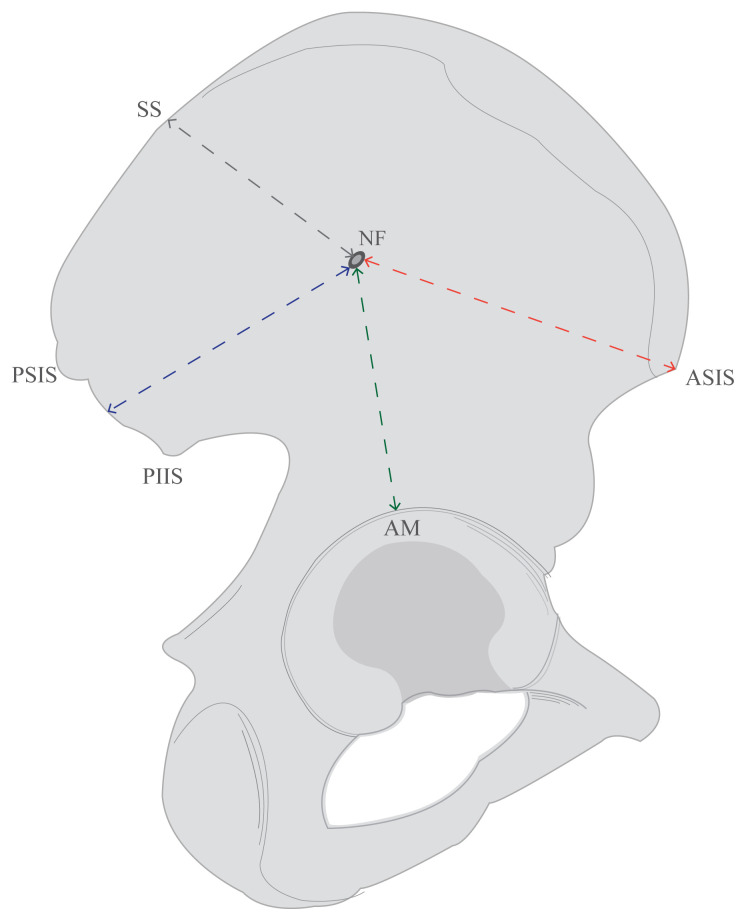
Illustration of measurements on the external surface of the hip bone. (NF: Nutrient foramen; SS: Most superior point of sacropelvic surface; PSIS: Posterior superior iliac spine; PIIS: Posterior inferior iliac spine; ASIS: Anterior superior iliac spine; AM: Acetabular margin; Black line: Distance between foramen and the most superior point of Sacropelvic Surface (SS); Blue line: Distance between foramen and midpoint of the line between PSIS-PIIS; Green line: The nearest distance between foramen and acetabular margin; Red line: Distance between foramen and ASIS).

The diameters of the foramina and their nearest distances to certain anatomical landmarks were measured, which were the acetabular margin, the most superior point of sacropelvic surface, midpoint of the line between the posterior superior iliac spine and posterior inferior iliac spine, and anterior superior iliac spine (Figure 2). Anatomical points and external bony landmarks that are easy to evaluate during surgery were chosen to determine the regions, where the foramina are frequently found [14]. Foramina that were greater than 1.5 mm in diameter on the external surface, were defined as principal foramina.

### 2.2. Internal surface

The foramina on the internal surface were evaluated according to whether they were above or below the pelvic brim. The configurations of the foramina were recorded. Diameters of all nutrient foramina were measured. Foramina that were less than 1 mm in diameter were not included in the evaluation.

**Figure 3:**
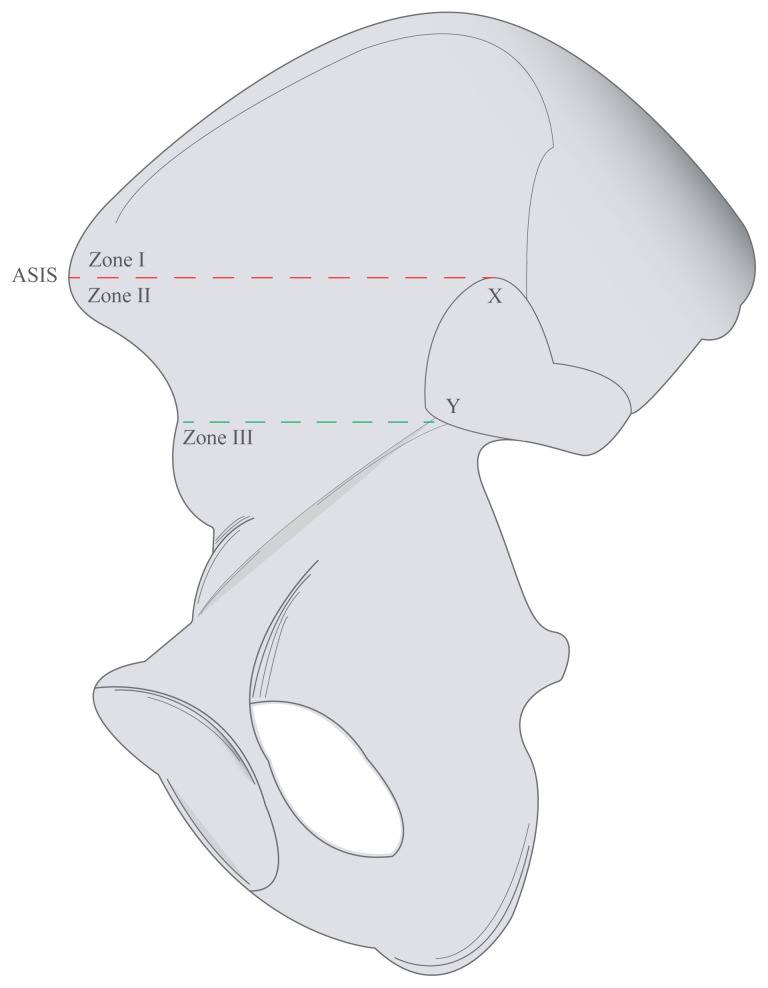
Illustration of 3 regions on the internal surface according to 2 parallel lines. (ASIS: Anterior superior iliac spine; X: Most superior edge of the auricular surface; Y: Edge of the auricular surface intersecting the pelvic brim; First line (red): Between the most superior edge of the auricular surface and anterior superior iliac spine; Second line (green): Parallel to the first (red) line starting from the edge of the auricular surface intersecting the pelvic brim; Zone I: The area above the first (red) line; Zone II: The area between two lines; Zone III: The area below the second (green) line).

The internal iliac surfaces above the pelvic brim were divided into 3 regions, via 2 parallel lines; the first line extends between the most superior edge of the auricular surface (X) and the anterior superior iliac spine; the second line extends parallel to the first line starting from the edge of the auricular surface which is intersecting the pelvic brim (Y) (Figure 3). Three zones were defined according to these two lines; the area above the first line as Zone I, the area between two lines as Zone II and the area below the second line as Zone III (Figure 3). In the literature, ASIS was preferred as the entry point during dissection to the iliac fossa [14], and also X and Y points are used to determine the risk areas during the operative fixations of the anterior sacroiliac joint in accordance with the literature [13,14].

**Figure 4:**
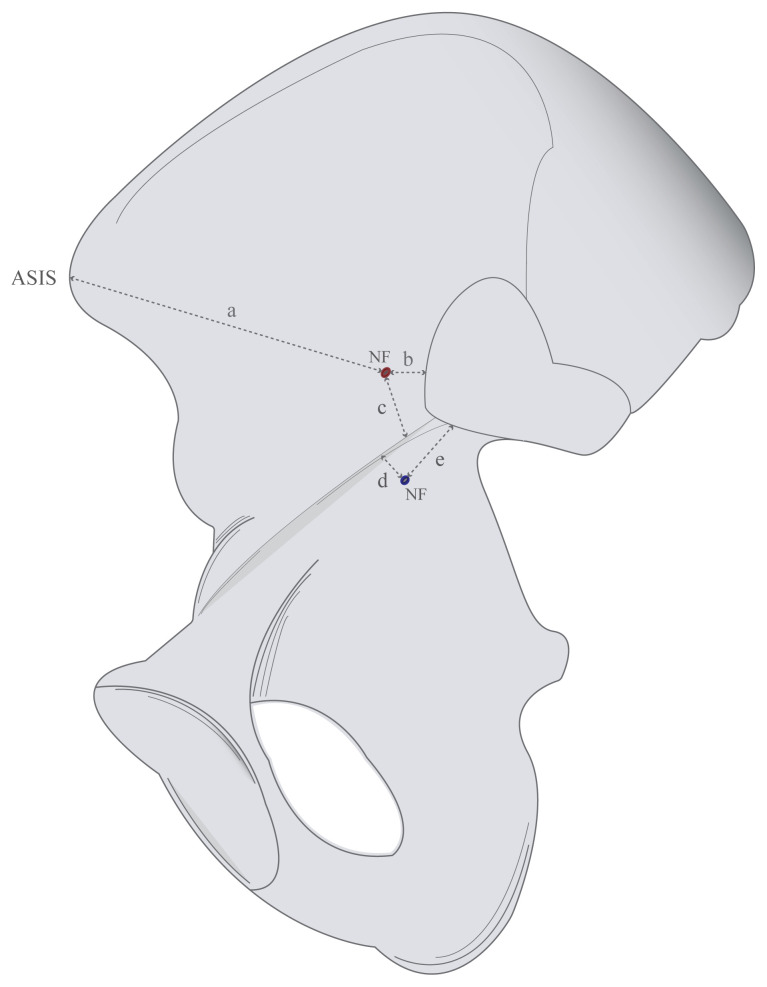
Illustration of the measurements on the internal surface (NF: Nutrient foramen; ASIS: Anterior superior iliac spine; a: Distance between foramen and ASIS; b: The nearest distance between foramen and anterior border of auricular surface; c: The nearest distance between foramen and pelvic brim; d: The nearest distance between foramen and pelvic brim; e: The nearest distance between foramen and inferior edge of auricular surface).

The quantities and localizations of nutrient foramina found on the internal surface were evaluated and grouped according to whether they were above (Group A) or below (Group B) the pelvic brim. The nearest distances of Group A to the anterior border of the auricular surface, anterior superior iliac spine, and pelvic brim [10,13]; and the nearest distances of Group B to the pelvic brim and the inferior edge of the auricular surface were measured (Figure 4). These measurements are important to determine the risk area for the application of the omega plate which is used in stabilization of pelvic ring and acetabular fractures [15].

Measurements were performed with a digital caliper with 0.01 mm accuracy by two independent anatomists. The approximate localizations of all foramina were indicated on the illustration with ImageJ software (National Institutes of Health, USA, http://rsbweb.nih.gov/ij/).

Statistical analyses were performed using the SPSS software version 23 (Statistical Package for the Social Sciences, IBM). Whether the data showed normal distribution was examined using histogram graphics and Kolmogorov-Smirnov/Shapiro-Wilk’s tests. The descriptive statistics of the data were given as mean, standard deviation, minimum and maximum values. The Mann-Whitney U test was used to compare the differences between the above and below internal surfaces where the foramen is located and diameters of the foramina because parametric test assumptions were not provided. A p-value of <0.05 was considered to be statistically significant.

## 3. Results

### 3.1. External surface

Fifty-one of 57 hip bones’ external surfaces were evaluated and 113 principal nutrient foramina were examined in total. Of the 51 bones, quantities of the detected nutrient foramina were 1 in 12 (23.53%), 2 or 3 in 36 (70.58%), 4 in 1 (1.96%) and 6 in 1 (1.96%). Unexpectedly no foramen was detected on one of the hip bones.

**Figure 5:**
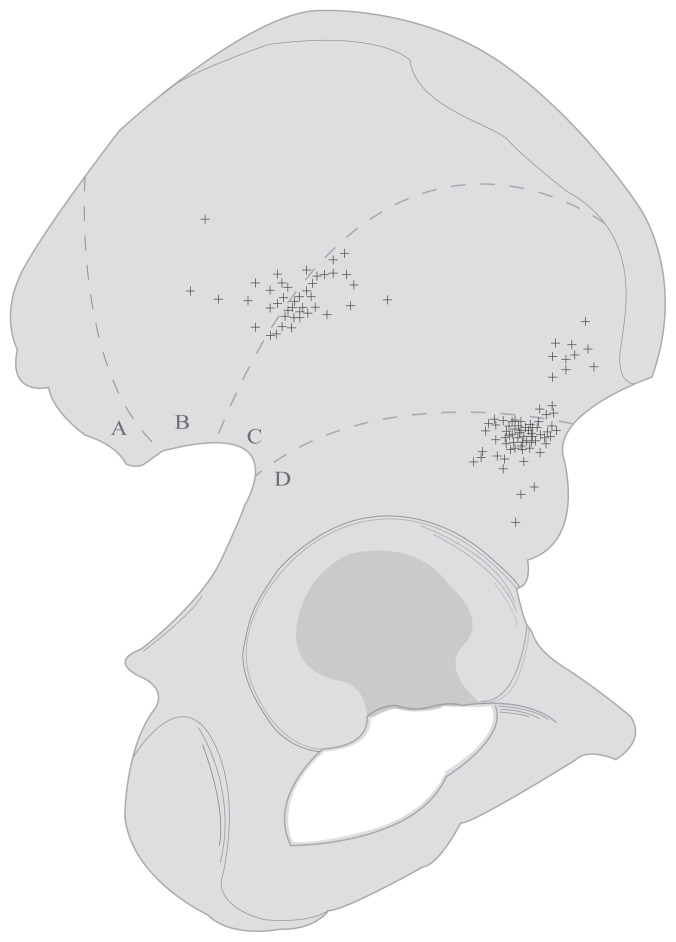
Illustration of the approximate localizations of the nutrient foramina of the external surface.

The localizations of the principal nutrient foramina were evaluated and 14 (12.39%) in Region B, 42 (37.17%) in Region C, and 57 (50.44%) in Region D were found. No principal nutrient foramen was found in Region A. The foramina in region C were located generally close to Regions B and D, and therefore they were evaluated together with these regions. According to the relations with Regions B and D, the foramina in Region C were divided into two groups as C-I and C-II respectively. Foramina in Regions B and C-I, and foramina in Regions D and C-II were evaluated together. The approximate localizations of all nutrient foramina were illustrated in Figure 5. The configurations of the foramina were evaluated as 63.72% round and 36.28% oval. The diameters of the nutrient foramina and their distances to the anatomical landmarks are shown in Table 1.

### 3.2. Internal surface

The principal nutrient foramina were most commonly located near the sacroiliac joint. The nutrient foramina on the internal surface of hip bones were evaluated according to whether they were above or below the pelvic brim.

**Figure 6:**
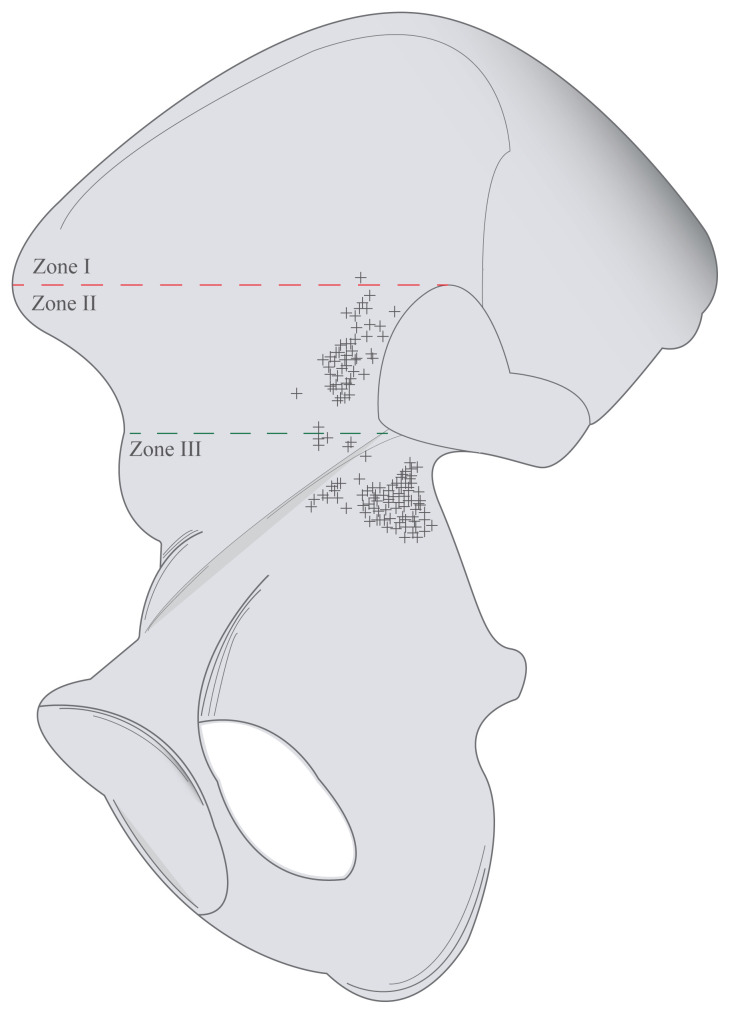
Illustration of the approximate localizations of the nutrient foramina of the internal surface above and below the pelvic brim.

A total of 54 nutrient foramina were found above the pelvic brim of the internal surfaces of all hip bones. Thirty-eight (66.66%) hip bones had only 1 foramen. Principal nutrient foramina were not observed in 11 hip bones (19.30%) and also there were no accessory foramina in 4 of them either. Moreover, 8 (14.04%) hip bones had 2 foramina. The localizations of the nutrient foramina were determined as 48 (88.89%) in Zone II, 5 (9.26%) in Zone III, and 1 (1.85%) in Zone I (Figure 6). The configurations of these foramina were 85.19% oval and 14.81% round.

There was a total of 81 nutrient foramina found below the pelvic brim (Figure 6). More frequently, 1 (43.86%) or 2 (38.60%) nutrient foramina were found in this area. Besides, 3 foramina were observed in 4 hip bones (7.01%). While principal nutrient foramen was not detected in 6 hip bones (10.53%), accessory foramina were. The configurations were 74.07% round and 25.93% oval.

The diameters of the nutrient foramina on the internal surface and their distances to certain anatomical landmarks are shown in Table 2. According to the results of the Mann-Whitney U test, there was no statistical difference between the diameters of the foramen above and below the pelvic brim on the internal surface (p = 0.212). The mean diameters according to the regions are shown in Table 3.

## 4. Discussion

Both the arterial blood supply and nutrient foramina of the ilium are not well described in the literature. While the current reports described only the area above the pelvic brim of the internal surface of the hip bone [10,13], no reports were found related to the external surface and the area below the pelvic brim on the internal surface of the ilium. Due to this missing information in the literature, this study was planned to describe the morphometric features of nutrient foramina found on the internal surface according to the pelvic brim and the external surface of the ilium. In this study, 57 hip bones’ internal surfaces were studied and 54 foramina were found above the pelvic brim and 81 foramina below the pelvic brim. In addition, 113 foramina were found on the external surface.

During the radiographic evaluations of the hip bone, nutrient foramina can be misinterpreted as stellate fractures or metastasis of malignancies [10,16–19]. Knowledge of the localizations and quantities of nutrient foramen should be kept in mind in the differential diagnosis of trauma patients [7,10].

The principal nutrient foramina on the internal surface are located generally 1 cm above the iliopectineal line and 1 cm anterior to the auricular surface. The nutrient branches of the iliolumbar artery enter the bone through this foramen [10,19,20]. Furthermore, the principal nutrient foramen is located anterior to the anterior gluteal line and branches of the superior gluteal artery enter the bone through this foramen on the external surface of hip bone [4,20]. It has been reported that foramina with smaller diameters are located below the iliopectineal line, in other words pelvic brim, and in front of the greater sciatic notch, and branches of the obturator artery enter the hip bone through those foramina [10,19,20].

Beck et al. studied 16 fresh frozen cadavers’ hip bones for evaluation of vasculature of the acetabulum and also the blood supply of pelvis on additional 4 hips. They reported that the nutrient arteries enter the bone in two different locations, according to their distance to the pelvic brim, 1 cm medial in 2 bones and 1 cm lateral in 2 bones [21].

Damaging of the nutrient artery located at the medial of pelvic brim may cause avascular necrosis of acetabulum in surgical procedures [19,21]. For instance, Šrám et al. reported that 2 of the 15 omega plate applications to the medial part of the pelvic brim resulted in avascular necrosis as a complication [15]. In terms of definition, unlike the other reports, these locations were defined as “above” instead of lateral and “below” instead of medial in this study.

In the literature, few studies have described the risk areas and safe zones for typical plate application of anterior approaches of internal fixations and open reductions of sacroiliac joints [13]. Nutrient arteries entering the foramina can be damaged by a fracture or surgical procedures [1]. Bleeding usually occurs from the nutrient foramina located near the sacroiliac joint [22]. Knowledge of the localization of the nutrient foramina can help to minimize intraoperative bleeding and prevent delayed union or nonunion problems in the postoperative period. Cao et al. reported that the bleeding during surgical procedures may require an average of 1200 mL blood transfusion [23]. It is generally necessary to apply bone wax to the nutrient foramen for homeostasis during intraoperative bleeding [20,24]. Also, temporal local packing or artery cauterization can be applied [14].

In this study the configurations of the nutrient foramina were studied on both external and internal surfaces, additionally, foramina that were found on the internal surface were defined according to the pelvic brim. The configurations of the foramina on the internal surface above the pelvic brim were reported in the literature and also in this study they were defined as 46 (85.19%) oval and 8 (14.81%) round. Ebraheim et al. also examined the configurations of 30 foramina on the internal surfaces but only the ones found above the pelvic brim and they reported that 7 of them were oval and 23 were round (Table 4) [10]. The differences between configurations were thought to be due to the course of the arteries.

Diameters of the foramina were also evaluated on both surfaces. While the mean values of the diameters of the foramina located above the pelvic brim on the internal surface were found to be 1.81 ± 0.54 mm in this study, Ebraheim et al. [10] similarly reported that the mean diameters of the foramina as 1.8 ± 0.4 mm.

In this study, the sacroiliac joint, pelvic brim, and anterior superior iliac spine were used as anatomical landmarks in accordance with the literature and the distances between foramina above the pelvic brim and these landmarks were measured [10,13]. The mean values were reported as 13.12 ± 4.22 mm, 19.88 ± 7.85 mm, and 83.46 ± 7.48 mm, respectively. Ebraheim et al. reported the mean distance between foramina and the sacroiliac joint as 12.5 ± 2.7 mm and Alla et al. as 18 mm. Ebraheim et al. reported the distance between the pelvic brim and the foramina as 23.5 ± 5.8 mm and Alla et al. as 20.1 mm [10,13]. Alla et al. also reported the distance between the anterior superior iliac spine and the foramina as 88.1 mm [13]. The knowledge of these distances between nutrient foramina and certain anatomical landmarks can reduce vascular complications. All comparisons are shown in Table 4.

Besides the knowledge of the vasculature of the pelvis, also the definition of the risk area for the anterior pelvic approach is important. Alla et al. defined the risk area according to the distances between nutrient foramina and certain anatomical landmarks as mentioned above for operative fixation of the anterior sacroiliac joint [13]. The “at- risk area” was identified as 80 mm to 95 mm medial to ASIS, 16 mm to 30 mm above the pelvic brim, and 12 mm to 25 mm lateral to SI joint. Additional caution should be taken to 80 mm medial to ASIS and 16 mm above the pelvic brim [13]. In this context, the at-risk area of this study was defined as 75–91 mm to ASIS, 12–28 mm to the pelvic brim, and 8– 18 mm to SIJ. The results were found to be compatible with the literature.

In this study, no foramen was detected on the external surface of one of the hip bones. Principal nutrient foramina were not observed on the above the pelvic brim of the internal surface of 11 hip bones (19.30%) and also there were no accessory foramina in 4 of them. While principal nutrient foramen was not detected below the pelvic brim of the internal surface in 6 hip bones (10.53%), accessory foramina were. Murlimanju et al. have stated these foramina may ossify with aging [11]. However, this could not be evaluated because the ages of the bones used in this study were unknown.

The limitations of this study are that it was performed with dry bones with unknown age and gender, and the sample size is limited. Also, vascular structures could not be evaluated.

## 5. Conclusion

The number and diameter of nutrient foramina may differ in each individual bone. Knowledge of the localization of the nutrient artery and foramina can avoid vascular complications in the anterior approach of the sacroiliac joint. This will help a better outcome of the surgical procedures. The most significant characteristic of this study is that all nutrient foramina on all surfaces were evaluated morphometrically for the first time in the literature.

## Figures and Tables

**Table 1:** The diameters of nutrient foramina and distances to certain anatomical landmarks.

REGIONS	Diamater of the foramen Mean ± SD (min-max) (mm)	Acetabular margin Mean ± SD (min-max) (mm)	Most superior point of sacropelvic surface Mean ± SD (min-max) (mm)	PSIS-PIIS midpoint Mean ± SD (min-max) (mm)	ASIS Mean ± SD (min-max) (mm)
**A** (n = 0)	-	-	-	-	-
**B** (n = 14)	2.26 ± 0.55 (1.54 – 3.55)	75.88 ± 7.93 (64.35–89.40)	47.75 ± 8.71 (33.60–65.02)	61.35 ± 10.36 (46.38–78.60)	91.99 ± 13.58 (72.39–116.85)
**C** (n = 42)	2.22 ± 0.47 (1.50–3.42)	57.18 ± 14.13 (26.67–81.52)	79.15 ± 25.84 (49.60–129.35)	86.64 ± 22.41 (54.78–123.27)	60.47 ± 23.60 (19.04–99.25)
**C-I** (n = 28)	2.30 ± 0.5 (1.56–3.42)	65.29 ± 7.47 (54.23–81.52)	62.02 ± 8.16 (49.60–75.35)	72.13 ± 9.36 (54.78–97.24)	75.84 ± 9.93 (55.31–99.25)
**C-II** (n = 14)	2.06 ± 0.39 (1.50–2.69)	40.95 ± 9.42 (26.67–60.82)	113.42 ± 8.47 (102.33–129.35)	115.67 ± 6.35 (102.42–123.27)	29.72 ± 4.99 (19.04–38.99)
**D** (n = 57)	2.58 ± 0.59 (1.54–4.08)	23.42 ± 3.72 (11.77–30.57)	112.79 ± 7.52 (93.76–127.94)	106.67 ± 12.03 (123.99–86.45)	41.95 ± 6.03 (31.18–52.60)
**B and C-I** (n = 42)	2.29 ± 0.51 (1.54–3.55)	68.82 ± 9.06 (54.23–89.40)	57.26 ± 10.69 (33.60–75.35)	68.54 ± 10.87 (46.38–97.24)	81.23 ± 13.52 (55.31–116.85)
**D and C-II** (n = 71)	2.48 ± 0.59 (1.50–4.08)	26.88 ± 8.76 (11.77–60.82)	112.92 ± 7.66 (93.76–129.35)	107.60 ± 8.34 (86.45–123.99)	39.54 ± 7.60 (19.04–52.60)

**Table 2:** The configurations, mean diameters, and distances to certain anatomical landmarks of the nutrient foramina on the internal surface.

	Oval	Round	Diameter of the foramen Mean ± SD (min-max) (mm)	Distance to the sacroiliac joint Mean ± SD (min-max) (mm)	Distance to the pelvic brim Mean ± SD (min-max) (mm)	Distance to the ASIS Mean ± SD (min-max) (mm)
**Above the pelvic brim** (n = 54)	46	8	1.81 ± 0.54 (1.20–3.36)	13.12 ± 4.22 (5.66–23.61)	19.88 ± 7.85 (1.16–39.58)	83.46 ± 7.48 (61.09–95.97)
**Below the pelvic brim** (n = 81)	21	60	1.66 ± 0.40 (1.20–3.05)	26.34 ± 8.90 (9.22–46.03)	18.17 ± 4.30 (8.62–28.07)	-

**Table 3:** The mean diameters according to the regions.

Foramen diameter by regions (Mean ± SD) (mm)	
**External surface**	2.41 ± 0.57
**Internal surface above the pelvic brim**	1.81 ± 0.54
**Internal surface below the pelvic brim**	1.66 ± 0.40

**Table 4:** Comparisons of morphometric measurements of nutrient foramina above the pelvic brim.

	Oval	Round	Diameter of the foramen Mean ± SD (mm)	Distance to the sacroiliac joint Mean ± SD (mm)	Distance to the pelvic brim Mean ± SD (mm)	Distance to the ASIS Mean ± SD (mm)
**Ebraheim et al.** (n = 30)	23	7	1.8 ± 0.4	12.5 ± 2.7	23.5 ± 5.8	-
**Alla et al.** (n = 11)	-	-	-	18	20.1	88.1
**Present study** (n = 54)	46 (85.19%)	8 (14.81%)	1.81 ± 0.54	13.12 ± 4.22	19.88 ± 7.85	83.46 ± 7.48
